# White matter microstructure differences between 15q11.2 copy number variation carriers and non-carriers in mid-to-late life

**DOI:** 10.1038/s41398-026-03962-2

**Published:** 2026-03-19

**Authors:** Max Korbmacher, Rune Boen, Ole A. Andreassen, Lars T. Westlye, Ida E. Sønderby, Ivan I. Maximov

**Affiliations:** 1 Neuro-SysMed Center, Department of Clinical Medicine, University of Bergen, Bergen, Norway; 2 Department of Health and Functioning, Western Norway University of Applied Sciences, Bergen, Norway; 3 Mohn Medical Imaging and Visualisation centre, Department of Radiology, Haukeland University Hospital, Bergen, Norway; 4 Semel Institute for Neuroscience and Human Behavior, Department of Psychiatry and Biobehavioral Sciences, University of California Los Angeles, Los Angeles, California USA; 5 Center for Precision Psychiatry, University of Oslo and Oslo University Hospital, Oslo, Norway; 6 K.G. Jebsen Centre for Neurodevelopmental disorders, University of Oslo, Oslo, Norway; 7 Department of Psychology University of Oslo, Oslo, Norway; 8 Department of Medical Genetics, Oslo University Hospital and University of Oslo, Oslo, Norway

**Keywords:** Neuroscience, Genetics

## Abstract

The 15q11.2 BP1-BP2 copy number variant (CNV) has been associated with neurodevelopmental and psychiatric conditions and brain grey matter structure, but its effects on white matter microstructure (WMM) in mid-to-late adulthood to assess long-term neurobiological effects remain unclear. Understanding these effects is important for evaluating long-term neurobiological impacts across the lifespan. WMM parameters were extracted from UK Biobank diffusion magnetic resonance imaging data for 15q11.2 BP1-BP2 deletion (n = 126, mean age: 66 ± 8) and duplication (n = 131, mean age: 64 ± 7) carriers, as well as age- and sex-matched non-carriers (n = 1260 and n = 1310). We used multiple advanced diffusion approaches, extending beyond DTI, providing metrics on various spatial levels comparing the CNV carriers and non-carriers. All metrics were projected on the WM skeleton for further group comparisons. We present various atlas-derived region-level differences between 15q11.2 BP1-BP2 deletion carriers and non-carriers. These differences suggest altered microstructural organization in the corpus callosum, cingulum and hippocampus, uncinate fasciculus, indicated by lower diffusivity and higher fractional anisotropy, kurtosis, axonal water fraction, and intra-neurite volume fraction (absolute standardized effects > 0.22). Most significant differences across multiple diffusion approaches were detected in the corpus callosum. Our findings suggest that during mid- to-late life, WMM in the corpus callosum is affected by 15q11.2 deletion. Different diffusion approaches allowing for microscopic assessments of WM, beyond previous non-microscopic diffusion MRI based assessments, suggest altered axonal density or microstructural organization, potentially reflecting pathological axonal overgrowth and myelination abnormalities, adding explanations for observable developmental and psychiatric differences between deletion carriers and healthy controls.

## Introduction

The 15q11.2 breakpoint BP1–BP2 copy number variant (CNV, i.e., deletion or duplication of genetic material at chromosome 15) is one of the most common recurrent CNVs [1], found in ~ 0.5% individuals in the population [2]. The 15q11.2 BP1-BP2 CNVs has been linked to an increased risk of neurodevelopmental and psychiatric conditions [3], including autism spectrum disorder, attention-deficit/hyperactivity disorder (ADHD) [2], schizophrenia [4, 5], and post-traumatic stress disorder [3], as well as cognitive deficits and dyslexia/dyscalculia. Additionally, several studies have identified group-level differences in CNV carriers’ brain structures when compared to non-carriers [6–10]. However, studies that comprehensively map the white matter microstructure (WMM) in 15q11.2 BP1-BP2 carriers are lacking. Here, we utilised several implementations of the standard diffusion model [11] of WM in the UK Biobank to assess the impact of the 15q11.2 BP1-BP2 CNV on WMM in mid-to-late life.

We focused on 15q11.2 BP1-BP2 CNV for several reasons: (1) it is one of the most common recurrent CNVs (~ 0.5% population frequency) [1, 2], enabling adequate sample sizes for neuroimaging analysis; (2) the genomic region contains genes (CYFIP1, NIPA1) with direct mechanistic links to axonal development and myelination, providing strong biological hypotheses for white matter alterations; (3) some previous diffusion tensor imaging (DTI) studies have reported white matter differences in this CNV [12, 13], which our advanced diffusion approaches can validate and extend; and (4) while penetrance for severe psychiatric disorders is indeed lower than some other CNVs, 15q11.2 deletion shows consistent associations with cognitive phenotypes (dyslexia, dyscalculia) and white matter-dependent functions.

The 15q11.2 BP1-BP2 genomic region contains four evolutionarily highly conserved genes: NIPA1, NIPA2, CYFIP1, and TUBGCP5. In relation to WMM, NIPA1 and CYFIP1 are of particular interest due to their role in axonal development. For instance, NIPA1 interacts with bone morphogenic protein (BMP) receptor type II to suppress BMP signalling, a pathway that plays a key role in axonal growth, guidance, and differentiation [14]. Moreover, CYFIP1 haploinsufficiency has been associated with myelin thinning in the corpus callosum in rodents [15, 16]. These associations could suggest that WMM might be affected in 15q11.2 CNV carriers. Mapping brain structural differences between CNV carriers and non-carriers might provide a further step towards mechanistic insights in the observed occurrence of psychiatric disorders among 15q11.2 CNV carriers.

By measuring molecular motion, diffusion magnetic resonance Imaging (MRI) offers unique possibilities to map the organisation of WM [11]. While changes in diffusion metrics are often interpreted as reflecting ‘structural integrity,’ in the context of neurodevelopmental risk variants, such alterations may reflect diverse pathological processes including aberrant axonal development, altered pruning, pathological overgrowth, or inflammatory changes. Previous DTI studies [12, 13] suggest that 15q11.2 BP1–BP2 deletion carriers exhibit widespread WM differences, primarily reflected by higher fractional anisotropy (FA) compared to non-carriers in several major tracts (i.e., higher FA in the anterior limb of the internal capsule, posterior limbs of the internal capsule and the hippocampal portion of the cingulum, lower FA in the posterior thalamic radiation). Other DTI metrics also revealed lower mean diffusivity in the body of the corpus callosum and the left uncinate fasciculus, as well as lower axial diffusivity in body and the splenium of the corpus callosum in deletion carriers vs controls. Moreover, WM alterations measured with DTI suggest opposing effects among duplication carriers compared to deletion carriers (incl. lower FA in the cingulum) [12, 13]. However, DTI metrics lack the spatial specificity to disentangle complex microstructural tissue properties, such as crossing or kissing fibres, potentially leading to inaccurate estimates of diffusivity [11]. Assessing WM on the microscopic level using advance diffusion MRI could reveal more biologically meaningful information on the (sub-)cellular structure instead of information on the scale of larger structures such as fibre bundles. This includes assessments of axonal properties in contrast to extra-axonal space, providing information on the actual WM-specific difference when comparing carriers to controls.

Hence, in this study, we provide the first comprehensive characterisation of WM at microscopic scale in 15q11.2 deletion and duplication carriers during mid-to-late life using advanced diffusion MRI approaches that extend beyond conventional scalar metrics from DTI. Our study focuses on mid-to-late adulthood, enabling the assessment of long-term neurobiological effects of genetic variation. We utilized different diffusion approaches allowing us to extract information on the microscopic level, including biologically meaningful estimates of axonal volume fractions, and intra- versus extra-axonal diffusivity. This diverse modelling framework in a well-characterised adult cohort provides novel insights into the long-term neurobiological impact of 15q11.2 CNVs on WMM and tests the robustness of previous carrier-control WM differences.

We utilized different diffusion approaches allowing us to extract information on the microscopic level, including biologically meaningful estimates of axonal volume fractions, and intra- versus extra-axonal diffusivity. This diverse modelling framework in a well-characterised adult cohort, our study provides novel insights into the long-term neurobiological impact of 15q11.2 CNVs on WMM and tests the robustness of previous carrier-control WM differences.

## Methods and materials

### Sample

We used UK Biobank [17] data from June 2023 (N = 45,196) with the retrieval procedure for neuroimaging data [18]. CNVs were based on the returned dataset from Crawford et al [19].

After quality control and overlapping the available T1-weighted and diffusion data with the Crawford dataset, we obtained a dataset of N = 37,083. Among these participants, data from deletion (n = 126) or 15q11.2 duplication (n = 131) carriers was included in the analysis. Additionally, each 15q11.2 deletion and duplication carrier was matched by age, sex, site, and intracranial volume with 10 control participants without any CNV (as defined by the returned dataset [19]) (n = 1310 and n = 1260; Table 1).

**Table 1:** Sample Demographics.

Deletion							
Variable	N	Mean	Std. Dev.	Min	Pctl. 25	Pctl. 75	Max
age	126	66	8.2	49	59	72	81
male sex	126	50%					
site	126						
… Cheadle	68	54%					
… Newcastle	33	26%					
… Reading	25	20%					
**Deletion matched controls**							
Variable	N	Mean	Std. Dev.	Min	Pctl. 25	Pctl. 75	Max
age	1260	66	7.6	47	60	72	82
male sex	1260	51%					
site	1260						
… Cheadle	712	57%					
… Newcastle	319	25%					
… Reading	229	18%					
**Duplication**							
Variable	N	Mean	Std. Dev.	Min	Pctl. 25	Pctl. 75	Max
age	131	64	7.1	49	60	69	81
male sex	131	44%					
site	131						
… Cheadle	70	53%					
… Newcastle	43	33%					
… Reading	18	14%					
**Duplication matched controls**							
Variable	N	Mean	Std. Dev.	Min	Pctl. 25	Pctl. 75	Max
age	1310	65	7.8	47	59	71	82
male sex	1310	43%					
site	1310						
… Cheadle	710	54%					
… Newcastle	425	32%					
… Reading	175	13%					

### Magnetic resonance imaging acquisition and processing

The MRI acquisition protocols for the UK Biobank can be found at https://www.fmrib.ox.ac.uk/ukbiobank/protocol/. After access to the raw diffusion MRI data, we processed the data using an optimised pipeline [20], including corrections for noise [21], Gibbs ringing [22], susceptibility-induced and motion distortions, and eddy current artifacts [23]. Isotropic 1mm^3^ Gaussian smoothing was carried out using *fslmaths* in FSL [24, 25] (version 6.0.1).

We used several diffusion approaches to extract WMM parameters derived from multi-shell data, including various diffusion approaches helped us to validate the potential findings from different approaches against each other and to extract nuanced spatial and structural information. These included, DTI [26], Diffusion Kurtosis Imaging (DKI) [27] and WM Tract Integrity (WMTI) [28] parameters were estimated using Matlab 2017b code (https://gitgub.com/NYU-DiffusionMRI/DESIGNER). Spherical Mean Technique (SMT) [29], and its multi compartment extensions (mcSMT) [30] parameters were estimated using original [29, 30] in-house C + + code (see https://github.com/ekaden/smt). The parameters for the Bayesian Rotationally Invariant Approach (BRIA) were evaluated with the original [31] Matlab code (https://bitbucket.org/reisert/baydiff/src/master/). For an overview, we processed 28 diffusion parameters, Supplemental Table 1. Two of the parameters (BRIA-DRAD extra and WMTI-axEAD) were excluded due to a high level of outliers and computational problems, resulting in 26 utilised metrics.

There are several reasons to estimate and present the parameters from multiple diffusion approaches. We included both DTI and DKI because DKI explicitly models non-Gaussian diffusion, which is ubiquitous in brain tissue, and provides more robust DTI estimates by accounting for kurtosis when fitting the tensor [32]. DTI and DKI are not redundant; rather, DKI-derived DTI metrics are methodologically superior to standard DTI. While SMT and DKI both extend beyond the tensor model, they do so with different assumptions: DKI is orientation-dependent and captures deviation from Gaussian diffusion, while SMT provides orientation-invariant estimates by powder-averaging. These complementary approaches test whether findings are robust across different modelling assumptions. Regarding crossing fibres: while our region-averaged approach does not explicitly model multiple fibre populations within voxels, SMT’s rotation-invariant framework is inherently less sensitive to crossing fibre confounds than DTI. Furthermore, our region-based averaging across multiple voxels partially mitigates crossing fibre effects that would be problematic in single-voxel interpretations. The utilised multi-compartment approaches further mitigate noise introduced by crossing fibres.

We used Tract-based Spatial Statistics (TBSS) [33], as part of FSL [24, 25]. Initially, all extracted fractional anisotropy (FA) maps were aligned to MNI space using non-linear transformation (FNIRT). Next, the mean FA image and mean FA skeleton were derived. Each diffusion scalar map was projected onto the mean FA skeleton using TBSS. To provide a quantitative description of diffusion parameters at the tract level, we used the John Hopkins University (JHU) atlas [34, 35] containing 20 tract averages based on a probabilistic WM atlas for each of the 26 parameters, totalling 520 values per individual. Additionally, we estimated the regional WM parameters for the 48 JHU regions for each of the 26 parameters, resulting in 1248 features per individual, as well as the whole skeleton average for each of the 26 parameters, totalling 1794 values per individual.

Quality control comprised of the YTTRIUM method [36], which converts global diffusion MRI scalar metrics into 2D format using a structural similarity extension of each scalar map to their mean image in order to create a 2D distribution of image and diffusion parameters. The YTTRIUM method used a structural similarity threshold of 0.85, with non-clustering values excluded, reducing the initial sample from N = 45,196 to N = 37,450. Additional exclusions entailed tract-based parameter outlier removal. Outliers were defined by exceeding 5 standard deviations from the mean, further reducing the sample from N = 37,450 to N = 37,083.

For the cortical reconstruction of T_1_-weighted data to obtain brain volumes, we used the FreeSurfer legacy version 5.3.0. Here, we only considered the total intracranial volume, which was used as a covariate.

Scanner ‘site’ in our analysis refers to the scanning location (Cheadle, Newcastle, Reading), which encompasses both scanner and location effects. All UK Biobank neuroimaging was performed on identical Siemens Skyra 3 T scanners with standardized protocols, minimizing scanner-specific variance. However, we harmonised datasets of duplication, deletion, and matched non-carriers independently using ComBat [37]. This strategy was chosen to avoid spillover effects from one cohort of interest to another, potentially inducing incorrect group differences through harmonisation [38], particular in the context of the small number of subjects per CNV group [39]. As a sensitivity analysis, we additionally harmonized all groups jointly. Primary results remained consistent across both harmonization strategies, with 89% of significant findings replicated.

### Statistical analyses

To validate against spatial and parcellation effects we conducted analyses averaging metrics on three spatial levels: global/whole-skeleton, tract, atlas-derived region. All statistical analyses were executed in R version 4.2.1 and used the same structure of a simple linear model predicting the outcome variable Y using age, age^2^, sex, the intracranial volume, and the respective grouping variable of carriers (deletion or duplication) vs non-carriers. We included the intracranial volume as it has previously been shown to distort diffusion estimates [40].

The Y was defined as global, tract, or JHU atlas-specific regional raw metrics, or principal components. For the principal components, we used the first component computed from a) tracts and b) JHU atlas-specific regions separately for each diffusion approach (DTI, DKI, etc.) specific collection of parameters.

To assess convergence across independent diffusion models, we computed Pearson correlation coefficients between parameters from the different diffusion approaches within the significant regions. This included comparisons of: DTI-FA vs SMT-FA, WMTI axonal water fraction vs SMTmc intra-neurite volume fraction, DTI mean diffusivity vs SMT mean diffusivity, and BRIA microscopic radial diffusivity vs WMTI radial extra-axonal diffusivity. Correlation matrices were generated separately for deletion carriers and matched controls.

To link microstructural differences to functional outcomes, we extracted relevant cognitive and behavioral phenotypes from UK Biobank for all participants. These included: reaction time (Field 20023, mean time to correctly identify matches), fluid intelligence score (Field 20016), symbol digit substitution test (Field 23324, processing speed measure), neuroticism score (Field 20127), and prospective memory (Field 20018). We correlated these measures with significant white matter metrics using linear models controlling for age, age², sex, and education level where available. Analyses were performed separately for deletion carriers, duplication carriers, and matched controls. False discovery rate correction was applied across all tests within each group.

As a sensitivity analysis step against spatial parcellations, to assess the overlap of significance across regions, we tested for carrier-control differences at the voxel-level using FSL randomise with 5000 permutations of non-parametric t-tests controlling for age, age^2^, sex, and intracranial volume for metrics highlighting significant group difference in the above-mentioned tests.

The significance level (alpha = 0.05) was corrected for all tests using the Benjamini-Hochberg false discovery rate (FDR) procedure. Correction was applied separately for each spatial scale to account for the hierarchical nature of our data: a) global analysis (skeleton-averaged metrics): 26 tests (26 diffusion metrics), b) tract-level analysis: 520 tests (26 metrics × 20 tracts), c) region-level analysis: 1248 tests (26 metrics × 48 JHU regions), d) PCA analysis: 12 tests (6 principal components × 2 spatial levels). Correction was performed separately for deletion and duplication comparisons, as these represent independent biological questions. This hierarchical approach controls Type I error rates while accounting for the nested structure of our data.

For voxel level assessments, we used threshold-free cluster enhancement [41] and family-wise error corrections to avoid false positives, which represents a separate multiple comparison correction from region-averaged analyses. All effects were standardised for comparability across scales.

## Results

None of the first principal components derived from tract- or region-level averaged WMM metrics presented significant differences between deletion or duplication carriers and non-carriers (p_FDR_ > 0.05, Supplemental files 1–4). Additionally, carrier-control comparisons of neither global, nor tract-level metrics between controls and carriers revealed no significant differences (Supplemental files 5–8).

Sensitivity analysis using joint harmonization across all groups confirmed the robustness of findings, with all but two significant regions (> 89% overlap) remaining significant after joint harmonization.

Correlation analyses revealed strong agreement between parameters from independent diffusion models within significant regions (Supplemental figs. 2–8). In the corpus callosum, DTI fractional anisotropy and SMT fractional anisotropy showed high correlation in both deletion carriers (r = 0.77) but not controls (r = 0.56), supporting convergent validity. WMTI axonal water fraction and SMTmc intra-neurite volume fraction demonstrated strong correlation in both deletion (0.93) and non-carriers (r = 0.90) indicating that independent biophysical models converge on consistent interpretations of tissue microstructure. Finally, DTI mean diffusivity and SMT mean diffusivity correlated at r = 0.93 in deletion carriers and at r = 0.90 in non-carriers. These high inter-model correlations (range: r = 0.77–0.93 in deletion carriers) demonstrate that findings are robust across different modelling assumptions and not artifacts of any single approach.

**Fig. 1:**
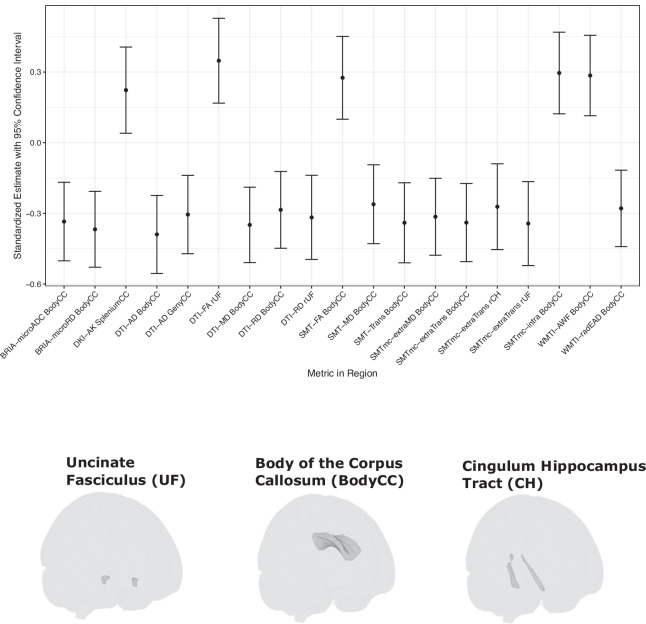
Regional white matter microstructure differences between 15q11.2 deletion carriers and non-carriers. Top panel: Significant Benjamini-Hochberg FDR-corrected (p_FDR_ < 0.05) standardized group differences (β-coefficients) across the John Hopkins University WM atlas regional metrics across diffusion approaches. Bottom panel: Visualisation of significant regions across metrics showing significant group differences. CC corpus callosum, CH = parahippocampal cingulum, r right, l = left. Displayed diffusion tensor imaging (DTI) measures: DTI-AD = axial diffusivity, DTI-MD = mean diffusivity, DTI-RD = radial diffusivity, DTI-FA = fractional anisotropy. Displayed kurtosis (DKI) metrics: DKI-AK = axial kurtosis. Displayed white matter tract integrity (WMTI) measures: WMTI-AWF = axonal water fraction, WMTI-radEAD = radial extra axonal water fraction. Bayesian rotationally invariant approach (BRIA) measures: BRIA-microADC = microscopic apparent diffusion coefficient, BRIA-microRD = microscopic radial diffusivity. Spherical mean technique (SMT) measures: SMT-FA = fractional anisotropy, SMT-MD = mean diffusivity, smt_trans = transverse diffusion coefficient. Multi compartment SMT (SMTmc) measures: SMTmc-extraMD. = extra-neurite microscopic mean diffusivity, SMTmc-extraTrans = extra-neurite microscopic transverse diffusivity, SMTmc-intra = intra-neurite volume fraction. For distributions of raw values for the presented group differences see Supplemental fig. 1. For correlations between diffusion models see Supplemental figs. 2–8. Significant test statistics can be found in Supplemental Table 2, and an overview of all conducted tests can be found in Supplemental file 10.

For region-level metrics, deletion carriers differed from non-carriers particularly in tracts stretching through the corpus callosum, parahippocampal cingulum, uncinate fasciculus, indicated by several significant group differences after corrections for multiple comparisons (Fig. 1, Supplemental Table 2). Metrics indicating altered microstructural organisation were different in deletion carriers compared to non-carriers (mean β = 0.29). These metrics included higher anisotropy- and kurtosis-related metrics, the axonal water and intra-neurite volume fractions. Concordantly, measures that indicate lower structural integrity, here, axial and radial diffusivity in different cellular compartments, were lower in deletion carriers (mean β = −0.32) across diffusion approaches.

The strongest group differences for diffusion metrics were found for DTI axial diffusivity (β = −0.39, 95% CI [−0.55, −0.22], p_FDR_ = 0.0001), WMTI microscopic radial diffusivity (β = −0.37, 95% CI [−0.53, −0.21], p_FDR_ = 0.0014), and DTI mean diffusivity (β = −0.35, 95% CI [−0.51, −0.19], p_FDR_ = 0.0018) in the body of the corpus callosum. Moreover, the strongest group differences for anisotropy and compartment-specific metrics incorporated DTI FA in the uncinate fasciculus (β = 0.35, 95% CI [0.17, 0.53], p_FDR_ = 0.0196), as well as SMTmc intra-neurite volume fraction (β = 0.30, 95% CI [0.12, 0.47], p_FDR_ = 0.0344) and WMTI axonal water fraction (β = 0.29, 95% CI [0.11, 0.46], p_FDR_ = 0.0472) in the body of the corpus callosum. Group-level summary statistics for the diffusion metrics can be found in Supplemental Tables 3–4.

For region-level metrics, duplication carriers did not differ from non-carriers (Supplemental file 9). This was reflected by the results from a sensitivity analysis of group-independently harmonised data (Supplemental files 11–15), as well as voxel-level analyses (Supplemental fig. 2), highlighting the identified corpus callosum carrier-control differences.

Post-hoc power analysis revealed that our duplication carrier sample (N = 131 carriers, N = 1310 controls) provided 81% power to detect effect sizes matching the median effect observed in deletion carriers (Cohen’s d = 0.31, calculated from standardized beta coefficients). Power was 78% for detecting medium effects (d = 0.30) but only 52% for detecting small effects (d = 0.20). The observed effect sizes in deletion carriers ranged from d = 0.22 to d = 0.39, with a median of d = 0.31. These power calculations indicate adequate power to detect medium-to-large effects, but limited sensitivity for small effects, suggesting that genuinely small duplication effects may have been missed rather than representing a true null finding.

## Discussion

This study provides the first characterisation of WMM in mid-to-late life carriers of 15q11.2 CNVs using a wide range of advanced diffusion MRI techniques, providing information of axonal structure on the microscopic level. While no significant differences were detected at the global or tract-averaged level, our region-specific analyses revealed consistent and sizable carrier-control differences in 15q11.2 deletion carriers but not in 15q11.2 duplication carriers. The effects identified were particularly pronounced in the corpus callosum, a region characterised by densely packed nerve fibres. Additionally, yet less consistent, certain diffusion metrics suggested differences in the uncinate fasciculus, parahippocampal cingulum, and hippocampal WM regions. Across multiple diffusion models, we found that 15q11.2 BP1-BP2 deletion carriers showed altered WM microstructural organisation compared to matched non-carriers, potentially reflecting aberrant axonal density, thickness or myelination. The observed pattern of microstructural differences in deletion carriers extends on and is consistent with prior DTI studies reporting elevated fractional anisotropy and reduced diffusivity in 15q11.2 deletions carriers [6–10].

Notably, the absence of consistent opposing effects in duplication carriers may suggest that a copy loss of 15q11.2 exerts more pronounced impacts on WM development than gene duplications. This is in line with the pattern of other CNVs where deletions carriers are often more affected than duplication carriers [10, 12, 42]. In comparison to a previous study [15] where participants were sampled from the UK Biobank, we observed fewer significant tracts of interest. This was also the case in comparison to the results obtained from an independent sample in another study [16]. Specifically, we only observed significant differences between 15q11.2 CNV deletion carriers and non-carriers when applying a region-wise JHU parcellation scheme. Yet, the magnitude and direction of the effects across studies align. Additionally, we apply a more stringent multiple testing threshold due to the use of additional diffusion approaches, which provide a more detailed description of white matter tissue.

Note also that it is crucial to interpret the observed microstructural patterns within the context of a neurodevelopmental risk variant. While lower FA and higher diffusivity has been documented as an effect of healthy neurodegeneration [43], what appears as ‘higher FA’ and ‘lower diffusivity’ should not be simplistically interpreted as ‘healthier’ or ‘better’ white matter in the present sample. In the context of 15q11.2 deletion, a known risk factor for neurodevelopmental conditions, these patterns may reflect pathological processes including: (1) aberrant axonal development with reduced pruning, leading to overcrowded axonal architecture; (2) pathological axonal overgrowth driven by altered BMP signalling (NIPA1 haploinsufficiency increases BMP, promoting axonal growth); (3) myelin abnormalities, as CYFIP1 haploinsufficiency has been linked to myelin thinning despite normal or increased axon numbers; or (4) altered tissue organization resulting from disrupted neurodevelopmental trajectories. Similar patterns of ‘increased FA’ have been reported in other neurodevelopmental conditions including autism spectrum disorder [44] or do not show differences in certain epilepsies [45], where they are interpreted as reflecting atypical neural architecture rather than enhanced function.

The observed pattern should not be interpreted as ‘healthier’ white matter, but rather as altered neurodevelopmental trajectory potentially reflecting pathological axonal overgrowth with concurrent myelination abnormalities.

The convergence of findings across multiple independent diffusion models, each with distinct underlying assumptions and sensitivities, substantially strengthens confidence that the observed alterations reflect genuine biological differences rather than model-specific artifacts. DTI provides foundational tensor metrics; DKI extends beyond Gaussian assumptions; WMTI decomposes signals into intra- and extra-axonal compartments; SMT offers rotation-invariant measures; SMTmc resolves microscopic tissue compartments; and BRIA provides Bayesian estimation of compartment-specific properties. The high correlations between conceptually related metrics in deletion carriers (r = 0.77–0.93) and consistent direction of effects across these diverse approaches support robust biological interpretation of corpus callosum alterations in deletion carriers.

Among the most robust findings in our study were differences in the corpus callosum, a structure critical for inter-hemispheric communication, indicating clear differences in microstructural organisation in 15q11.2 BP1–BP2 deletion carriers compared to noncarriers. The corpus callosum showed the strongest group effects, independent of assumptions about diffusivity in different tissue compartments (biophysical models). This was particularly the case for measures related to axonal density and restricted diffusion. These results are notable given the corpus callosum’s central role in neurodevelopment and its frequent implication in a range of psychiatric and neurodevelopmental conditions [46, 47]. While such observed differences are often interpreted to reflect altered axonal architecture, we cannot determine whether it is one or the other. However, haploinsufficiency of CYFIP1 has previously been suggested to contribute to thinner myelin in callosal fibres and reduced oligodendrocyte numbers. Thus, our findings may potentially reflect impaired myelination while axons are denser packed [48]. Moreover, downregulation of NIPA1 has been shown to increase BMP signalling [14], which can drive axonal overgrowth. Following from that, our results might reflect axonal overgrowth, which presents itself altered microstructural organisation measured by the examined microstructure metrics. The axonal overgrowth might coincide with reduced or immature myelination, leading to a denser yet less myelinated axonal architecture within the corpus callosum. Given that the corpus callosum is the most myelinated white matter structure in the human brain, this might provide the largest sensitivity for discovery of myelination-related abnormalities and thus would explain our observations there.

Axonal overgrowth, and hence measures suggesting altered axonal density in the corpus callosum is in line with previously observed larger corpus callosum volumes in 15q11.2 deletion carriers versus healthy controls [46, 49]. Additionally, axonal overgrowth in the thalami has previously been associated with the genetic risk for schizophrenia [50]. These previous findings suggest that the observed microstructural alterations in the corpus callosum might refer to an altered axonal metabolism or even generation of cells. The resulting effects on signal transmission might be expressed in impaired inter-hemispheric communication, posing a potential neurobiological process behind the mechanism of dyslexia which along with dyscalculia is more prominent among in 15q11.2 deletion carriers [46].

We also observed significant group differences in the uncinate fasciculus which has been implicated in emotion regulation and the risk for affective disorders such as major depressive and generalized anxiety disorders, previously correlated with lower fractional anisotropy [51–53]. An additional finding in this paper was alterations in the parahippocampal cingulum which is associated with ADHD [54]. Alterations in both tracts have also been linked to prenatal stress, depression, and anxiety [55], suggesting that WM changes in these pathways may contribute to lifelong psychological vulnerability or resilience. The direction of the effects reported in our study (higher FA and lower transverse diffusion coefficient in 15q11.2 BP1-BP2 deletion carriers compared to non-carriers for the corpus callosum, uncinate fasciculus and parahippocampal cingulum) may reflect altered microstructural organization. Indeed, the uncinate fasciculus and the parahippocampal cingulum tracts consist of fibre pathways that are part of limbic-frontal circuitry, which has been found to exhibit weaker functional connectivity in CYFIP1 haploinsufficient rats [48]. Nevertheless, the functional implications of the observed group differences remain unclear and warrant further investigation.

The absence of significant findings in duplication carriers, combined with adequate power (81%) to detect effect sizes matching the median deletion effect (d = 0.31), suggests several

possibilities: (1) duplication effects on white matter are genuinely smaller than deletion effects, consistent with broader CNV literature showing deletions typically exert stronger impacts than duplications; (2) duplication carriers may exhibit greater phenotypic heterogeneity, with some individuals showing alterations masked at the group level by substantial inter-individual variability; (3) compensatory mechanisms in duplication carriers may obscure microstructural changes that are detectable with current linear modelling approaches; or (4) the biophysical models employed may have differential sensitivity to the underlying pathophysiological processes in deletion versus duplication. Future studies with larger duplication samples or alternative analytical approaches (e.g., machine learning methods sensitive to multivariate patterns) may reveal effects not captured here. Nevertheless, the pattern of stronger deletion effects aligns with findings across multiple CNV studies, where copy number loss generally confers greater phenotypic impact than gain.

We acknowledge that 15q11.2 BP1-BP2 has lower penetrance for major psychiatric disorders compared to some other neurodevelopmental CNVs (e.g., 22q11.2 deletion, 16p11.2). However, this property also makes it valuable for studying brain alterations that may confer vulnerability without inevitably leading to clinical diagnosis. The participants in our study, who reached mid-to-late adulthood without severe psychiatric diagnoses, likely represent a relatively resilient subset of carriers, potentially with protective genetic or environmental factors that moderated CNV effects. This survival bias means our findings may underestimate alterations present in more severely affected carriers who may be underrepresented in large epidemiological cohorts. Future work extending this multi-modal diffusion approach to CNVs with higher penetrance and larger effect sizes (e.g., 22q11.2 deletion syndrome) would provide valuable comparisons and potentially reveal whether the microscopic white matter signatures we observe are specific to 15q11.2 or represent a common pathway across neurodevelopmental CNVs.

Some strengths of this study in comparison to previous WM studies on 15q11.2 [12, 13] is i) the use of several diffusion approaches, beyond DTI; ii) the signal decomposition that accounts for both kurtosis and conventional tensor imaging for DTI estimates, which leads to more robust and reproducible estimates of DTI estimates [32], iii) the harmonisation of data to control for non-biological effects induced by scanners or site-specific characteristics [12, 13], and the inclusion of relevant covariates such as intra-cranial volume which can bias the results [40].

Our results should not be interpreted without considering some limitations. First, despite our sample being among the largest to date for rare CNV carriers, statistical power remains limited for detecting subtle effects in duplication carriers. Second, while advanced diffusion approaches offer greater biological specificity than DTI, they remain indirect proxies of microstructure and are sensitive to modelling assumptions in the used approaches. Third, we acknowledge that the white matter alterations we observe in mid-to-late adulthood likely originated during neurodevelopment and have persisted across the lifespan. We cannot determine from cross-sectional data whether these represent stable developmental differences, progressive changes, or compensatory adaptations that emerged over time. The term ‘mid-to-late life’ refers to when we observe these differences, not necessarily when they arose. Additionally, our sample of carriers who reached late adulthood without severe psychiatric diagnoses likely represents a relatively resilient subset, potentially with protective genetic or environmental factors. This survival bias means our findings may underestimate alterations present in more severely affected carriers who may be underrepresented in epidemiological cohorts. Longitudinal studies beginning in childhood would be needed to characterize the developmental trajectory of white matter alterations in 15q11.2 carriers.

In conclusion, this study reveals region-specific WMM differences in 15q11.2 deletion carriers during mid-to-late adulthood, particularly in the corpus callosum, parahippocampal cingulum, hippocampal tracts, and uncinate fasciculus. These effects indicate altered microstructural organization in 15q11.2 BP1-BP2 deletion carriers compared to non-carriers, possibly reflecting altered axonal density or organization, extending prior findings and emphasizing the value of advanced diffusion imaging in uncovering the neurobiological impact of CNVs. Linking the examined structural alterations to cognitive and clinical phenotypes in CNV carriers might be useful future contributions, ideally in longitudinal designs that can assess how WM differences evolve with age.

## Supplementary information

Supplemental Figures

Supplemental File 1

Supplemental File 2

Supplemental File 3

Supplemental File 4

Supplemental File 5

Supplemental File 6

Supplemental File 7

Supplemental File 8

Supplemental File 9

Supplemental File 10

Supplemental File 11

Supplemental File 12

Supplemental File 13

Supplemental File 14

Supplemental File 15

Supplemental Table 1

Supplemental Table 2

Supplemental Table 3

Supplemental Table 4

## Data Availability

This study has been conducted using UKB data under Application 27412. All raw data are available from the UKB (www.ukbiobank.ac.uk). UK Biobank has approval from the North West Multi-centre Research Ethics Committee (MREC) as a Research Tissue Bank (RTB) approval. The raw and processed UK Biobank MRI data are protected and are not openly available due to data privacy laws. Unfortunately, the most recent changes in UKB policy urge us to delete all UKB data from our servers, limiting the computational reproducibility of our analyzes and future research based on our data. Updated procedures on how to obtain indirect data access against an access fee might be available on the UKB website (see https://www.ukbiobank.ac.uk/enable-your-research/apply-for-access).

## Code availability

Analysis scripts and Supplemental files, including 2D (tract- and region based) and untresholded 3D (voxel-based) summary stats can be found at https://github.com/MaxKorbmacher/15q11.2WhiteMatterMicrostructure.
