# Supplementary material for: White matter microstructure differences between 15q11.2 copy number variation carriers and non-carriers in mid-to-late life
Source: Transl Psychiatry. 2026 Mar 19;16:190. doi: 10.1038/s41398-026-03962-2 (PMC13039188; doi:10.1038/s41398-026-03962-2)
Supplement: Supplementary file 1 — Supplemental Figures [file 41398_2026_3962_MOESM1_ESM.docx]

**Supplemental Figures**

Supplemental figures for the article “White matter microstructure differences between 15q11.2 copy number variation carriers and non-carriers in mid-to-late life”, Korbmacher et al., 2025.

Supplemental tables can be found as separate files.

***Supplemental Figure 1.* Ridgeline plots of raw values for the deletion and the matched healthy control group which were highlighted as significant**

***Supplemental Figure 2.* Sagittal view of p<0.001 thresholded results for DTI-FA, SMT-FA, and SMTmc-intra as examples of voxel-level and region-level correspondence**

**
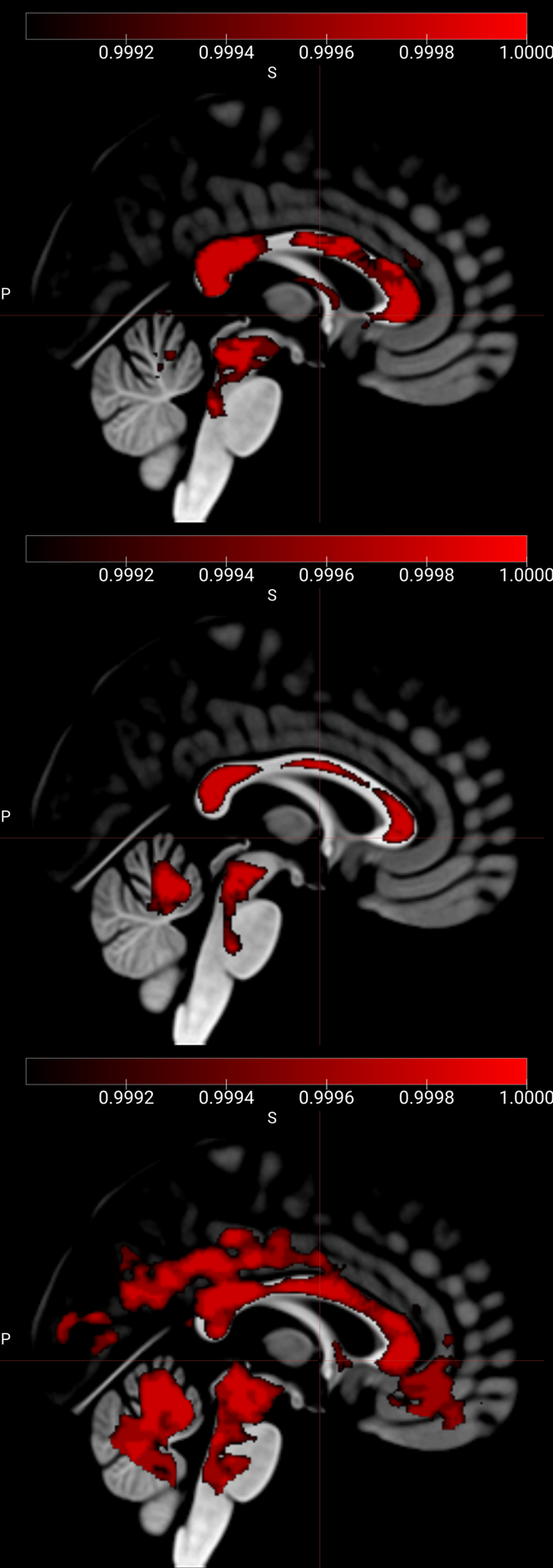
**

From top to bottom: DTI-FA, SMT-FA, and SMTmc-intra. Black to red values are presented for 1-alpha, where alpha = 0.001.

***Supplemental Figure 3.* Metric level correlations in the corpus callosum of deletion carriers**

***Supplemental Figure 4.* Metric level correlations in the splenium of the corpus callosum of deletion carriers**

***Supplemental Figure 5.* Metric level correlations in the uncinate fasciculus of deletion carriers**

***Supplemental Figure 6.* Metric level correlations in the corpus callosum of deletion carrier matched controls**

***Supplemental Figure 7.* Metric level correlations in the splenium of the corpus callosum of healthy controls matched to the deletion carrier**

***Supplemental Figure 8.* Metric level correlations in the uncinate fasciculus of healthy controls matched to deletion carriers**
